# Plant foods consumed at the Neolithic site of Qujialing (ca. 5800-4200 BP) in Jianghan Plain of the middle catchment of Yangtze River, China

**DOI:** 10.3389/fpls.2022.1009452

**Published:** 2022-10-18

**Authors:** Muslim Khan, Ling Yao, Yuzhang Yang, Yang Tao, Weiya Li, Dewei Zhang, Yunbing Luo, Juzhong Zhang

**Affiliations:** ^1^ Department for the History of Science and Scientific Archaeology, University of Science and Technology of China, Hefei, China; ^2^ Hubei Provincial Institute of Cultural Relics and Archaeology, Wuhan, China; ^3^ Key Laboratory for Archaeological Science and Cultural Heritage, Department of Education of Anhui Province, Hefei, China

**Keywords:** Jianghan plain, Qujialing, the late Neolithic, plant foods, starch grain analysis

## Abstract

The site of Qujialing experienced a long, sustained process of the development of Neolithic culture in the Jianghan Plain, with a period of some1600 years. Our previous studies based on macrofossil remains and phytoliths revealed that rice (*Oryza sativa*) from Qujialing was already domesticated, and millet (*Setaria italica* and *Panicum miliaceum*) had also been spread into the site since the Youziling Culture period (5800-5100 BP). Nevertheless, no direct evidence has been provided regarding the daily consumed plant foods, especially plant foods obtained by gathering, throughout the site occupation. This paper thus examines pottery sherds (n=41) associated with culinary practices from Qujialing with starch grain analysis. Apart from starch grains from rice and millet, the results indicate that job’s tears (*Coix lacryma-jobi*), lotus roots (*Nelumbo nucifera*), tubers possibly from Chinese yam (*Dioscorea panthainca*), acorns (*Quercus* sp.), and beans (*Vigna* sp. or/and *Vicia* sp.) were consumed by the ancient Qujialing people, within job’s tears and lotus roots were not discovered before in the macrofossil remains and phytoliths. Combining the starch data and multiple lines of evidence from macrofossil remains and phytoliths, it is suggested that rice was among the most frequently consumed plant foods since the first occupation phase at Qujialing, while acorns could have been gradually replaced by other agricultural products (i.e., rice) and became less important food ingredients, especially when agriculture was more developed in the last occupation phase at Qujialing. These novel findings not only complement our previous research by providing the first line of evidence of paleodiet in the Jianghan Plain from the perspective of starch grain analysis but also delivers a better understanding of the characterized dietary trends and agricultural development in the research region.

## Introduction

Early agriculture has been the subject of major interest for archaeologists over the past few decades, with much of the focus on the origins and spread of different agricultural products worldwide (Ammerman and Cavalli-Sforza, 1971; Bellwood, 2005; Fuller, 2011; Yang et al., 2016a; Luo et al., 2019b). China was one of the world’s oldest centres of independent agricultural development. The most thoroughly studied early agricultural societies in China are located along the Yangtze and Yellow River Valleys, which provide some of the earliest compelling evidence for rice (*Oryza sativa*) and millet (*Setaria italica* and *Panicum miliaceum*) cultivation accordingly (Jiang and Liu, 2006; Liu et al., 2007a; Liu et al., 2007b; Yang et al., 2012; Wu et al., 2014). The gradual transition from foraging to agriculture during the Neolithic period, also known as the “agricultural revolution”, forever changed how humans live, eat, and interact in southern and northern China. Since then, the human population was able to grow exponentially because crops and animals could be farmed to meet demand. This revolution also stimulated significant developments in social organization and technology, paving the way for the Chinese civilization process.

The research region in this paper, namely the Jianghan Plain situated in the middle catchment of the Yangtze River basin, is also a pivotal zone for studying rice domestication and the formation of mixed farming of rice and millet in early China. The Jianghan Plain is an alluvial plain, which was named for the confluence of the Yangtze and Han Rivers. It was once a large wetland but was gradually colonized by early farmers in the Neolithic period (Zhang, 2013). The Jianghan Plaine takes up most of central and southern Hubei Province in Central China, an area with a humid subtropical climate and four distinct seasons. The Neolithic Cultures that appeared in this region also played important roles in the origin and development of Chinese civilization (Fei, 2017). In the past 15 years, extensive work based on macrofossil remains and phytoliths has been carried out in the Jianghan Plain, especially at the archaeological sites attributing to the Youziling Culture period (5800-5100 BP), Qujialing Culture period (5100-4500 BP) and Shijiahe Culture period (4500-4200 BP) (Wu et al., 2010; Deng et al., 2013; Tang et al., 2014; Tang et al., 2017; Luo et al., 2019a; Yao et al., 2019; Yang et al., 2020). The yielded data consistently indicates that millet had already spread into the Jianghan Plain from northern China since the Youziling Culture period, but rice was still the most prominent crop. These accumulating archaeobotanical studies have provided vast information regarding prehistoric plant resources in the Jianghan Plain. However, previous research focused either on macrofossil remains or phytoliths, could not provide direct evidence regarding what was the daily consumed plant foods in the prehistoric farming communities. In addition, it should be noted that most phytolith research only analyzed the utilization of rice and millet, partially because many types of phytoliths have indistinct morphological differences from other types (Lu et al., 2009; Neumann et al., 2019). Moreover, considering the different taphonomy pathways of various plants, biases in macrofossil records were reported (Locatelli, 2014). For instance, underground storage organs (USOs) could have been consumed completely thus leaving few fossil remains, while husks of cereals, seeds of fruits, and shells of acorns might be preserved even after processing or consumption. It is also worth mentioning that, human skeletons and teeth were poorly preserved at the Neolithic sites in the research region for isotope or residue analysis to reconstruct the ancient diets at Qujialing. Under such circumstances, studies using another different analytical method (e.g., starch grain analysis) in the research region thus are imperative to provide more insights into the paleo diet in the Jianghan Plain.

**Figure 1:**
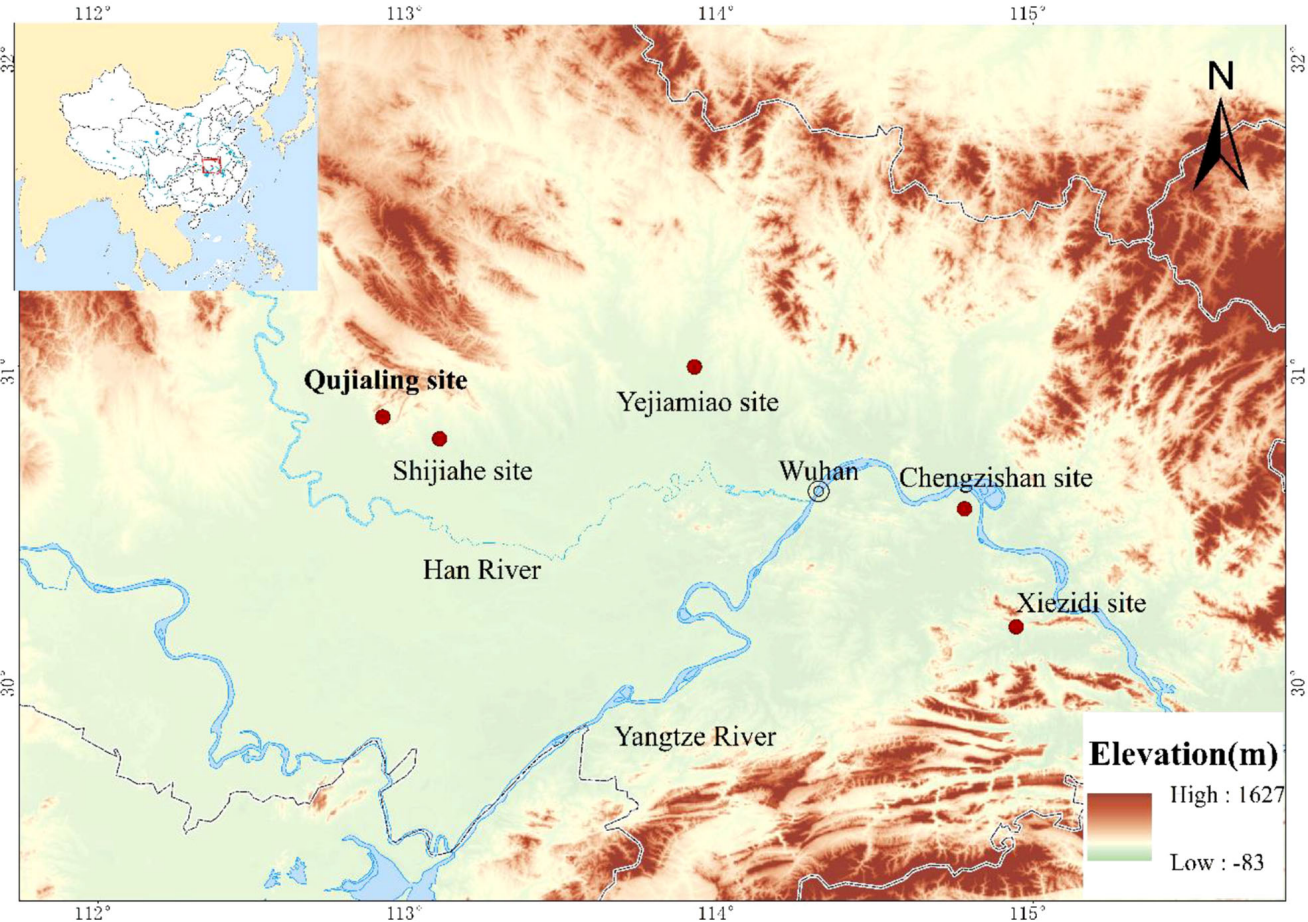
Locations of Qujialing and nearby sites in the middle catchment of Yangtze River.

**Figure 2:**
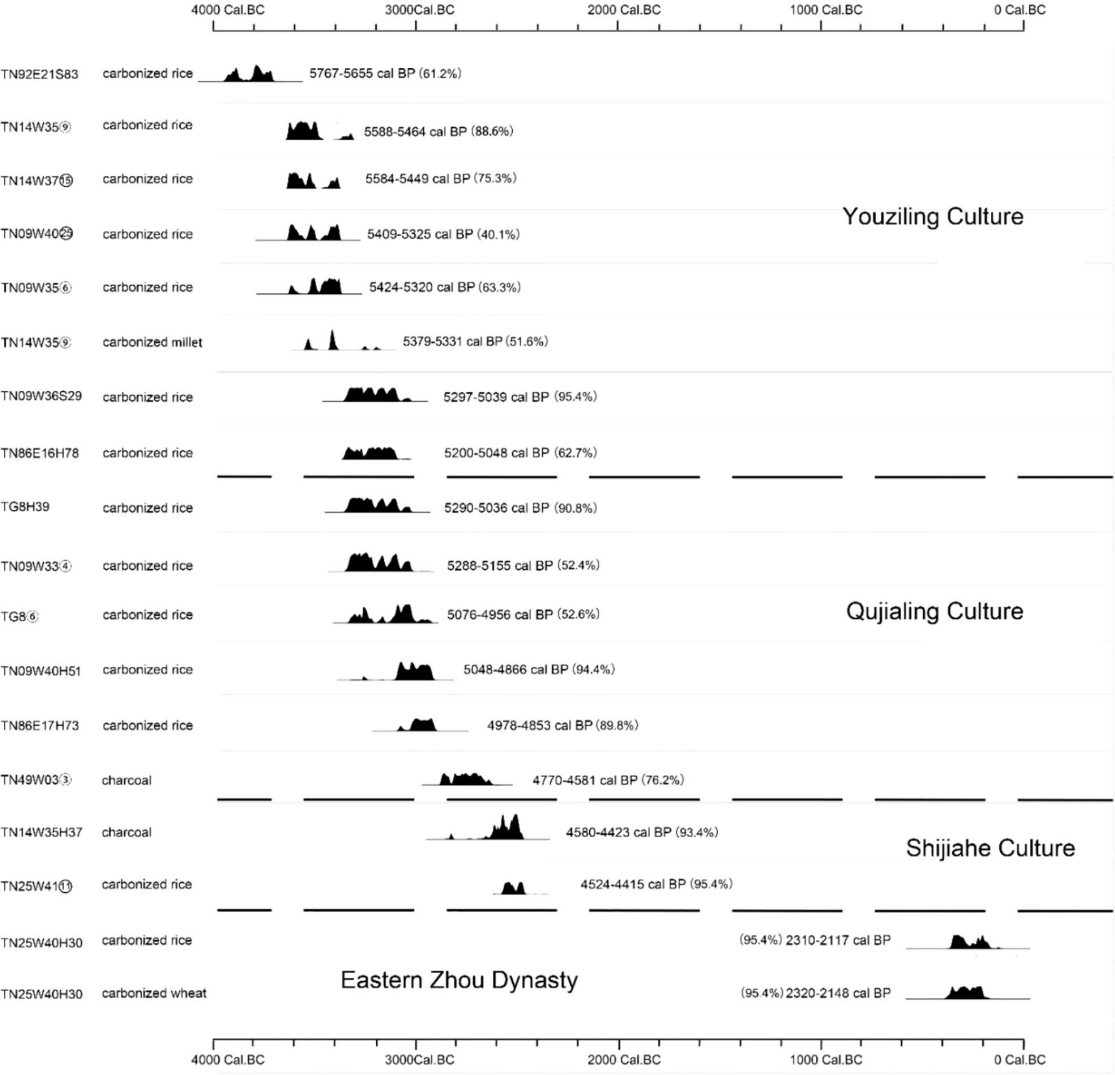
Carbon-14 dates and dendrochronologically corrected dates of charred macrofossil samples excavated at Qujialing (After Yao et al., 2019).

Qujialing (112°54’33.54’’E; 30°50’01.93’’N) is located in the east of the lower catchment of the Han River in Jingmen city, where is about 130 kilometers from Wuhan, the capital city of Hubei Province (**Figure 1**). The site has been recognized as one of the most representative settlement sites encircled by rivers in the Jianghan Plain, covers an area of 2.84 square kilometers (Tao et al., 2019). Qujialing was first discovered in 1954 and underwent its first excavation in 1955, which is also known as the first systematic excavation in the whole Hubei Province in China. In 1956, the second excavation at Qujialing brought striking examples of painted pottery vessels to light. Because of that, the Chinese archaeologists soon realized the importance of the site and named the Neolithic “Qujialing Culture” after the site of Qujialing. The finding of the Qujialing Culture, for the first time, unveiled the prehistoric culture with distinctive characteristics in the middle Yangtze River Basin. Since 1989, Qujialing went through another two excavation seasons (Tao et al., 2019; Wang et al., 2021), revealing the site had experienced an even longer and sustained process of development, from the Neolithic Youziling Culture period (Phase I) to the Qujialing Culture period (Phase II), and then to the Shijiahe Culture period (Phase III), with a period of approximately 1600 years (**Figure 2**). In addition, excavations at the site also discovered objects from the Eastern Zhou Dynasty (770-256 BP). In 2021, the site of Qujialing was nominated as one of the “Hundred Years of Archaeological Discoveries” in China, mostly because it witnessed the glorious stage of prehistoric cultural development in the middle catchment of the Yangtze River and its critical significance in tracing the origin and development of early Chinese civilization.

Our research team has been closely involved in the latest excavation at the site of Qujialing since the beginning, allowing us to conduct a more holistic archaeobotanical study at the site. In two of our previously published papers, macrofossil remains retrieved from flotation and phytolith remains discovered in the sedimentary soil samples from Qujialing have been studied (Yao et al., 2019; Yang et al., 2020), revealing rice, millet, and other types of plant species were available at the site (see more in the discussion section). Based on these findings, this paper further analyses pottery vessels that were associated with storing, cooking, and serving foods, using starch grain analysis. Although the different analytic method applied here has not been adopted for studying pottery vessels in the Jianghan Plain, it has been widely applied to objects that may have been in contact with starch-rich plants, such as lithic grinding tools, teeth (dental calculus), and pottery from elsewhere (e.g., Piperno et al., 2004; Lu et al., 2005; Buckley et al., 2014; Yang et al., 2016b; Li et al., 2020b; Liu et al., 2020). By recovering the preserved starch grains from ancient artefacts, this method can often identify the preserved starch remains to a genus taxonomic level and sometimes even species or subspecies level (Yang and Perry, 2013; Liu et al., 2014). The results, on one hand, will complement previous research based on macrofossil remains and phytoliths, providing more insights into the past exploitation of plants. On the other hand, the data will also answer what types of plant foods the ancient Qujialing people cooked and consumed throughout their occupation, thus enriching the discussion on how the paleo diet may have been shaped by the development of rice agriculture and the arrival of millet during the prehistoric period.

## Material and methods

**Figure 3:**
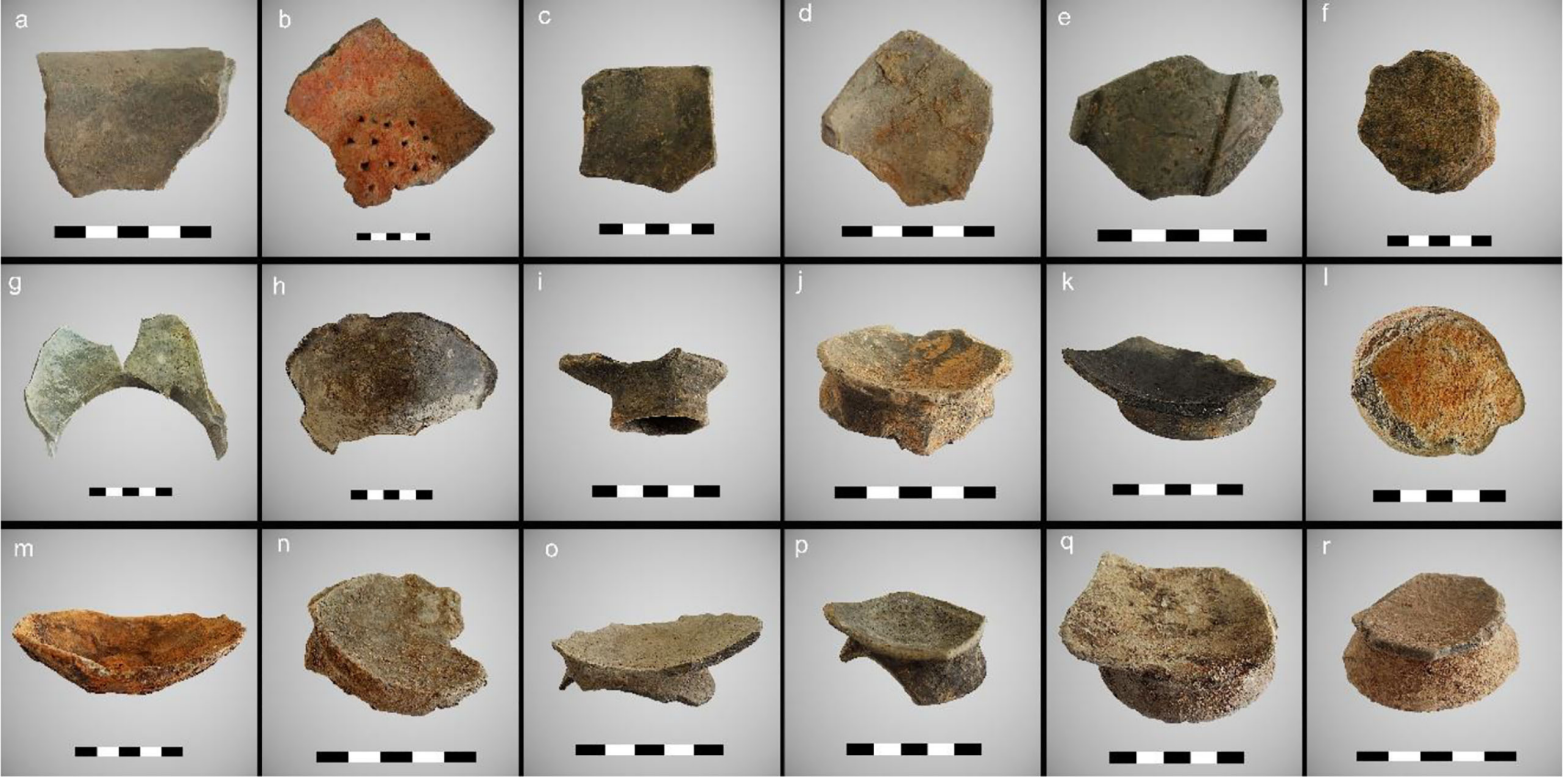
Examples of the analyzed pottery samples from the site of Qujialing (**A-F** are pottery sherds dated to the Youziling Culture period; **G-L** are pottery sherds dated to the Qujialing Culture period; m-r are pottery sherds dated to the Shijiahe Culture period; scale bar: 10cm **A**: 2016HQQTN11W30④: S26, **B**: 2016HQQTN10W30④: S15, **C**: 2016HQQTN10W30④: S12, **D**: 2016HQQTN11W30④: S27, **E**: 2016HQQTN10W30④: S10, **F**: 2016HQQTN10W30④: S9); **G-L** are pottery sherds dated to the Qujialing Culture period (**G**: 2017HQQTN49W03::S32-34, **H**: 2017HQQTN49W03::S94, **I**: 2016HQQTN10W30::S4, **J**: 2016HQQ ash pit, 2⑤:S155, **K**: 2016HQQ ash pit, 2⑤:S144, **L**: 2016HQQTN13W30::S40); **M-R** are pottery sherds dated to the Shijiahe Culture period) (**M**: 2016HQQTG8:: S75, **N**: 2016HQQTG8:: S76, **O**: 2016HQQTN25W41:S66, **P**: 2016HQQTN25W41:S63, **Q**: 2016HQQTN25W41:S62, **R**: 2016HQQTN09W39H29).

This paper studies 41 pottery sherds recovered from the latest excavation at the site of Qujialing between 2015 and 2017 (**Table 1**; **Figure 3**). Pottery sherds were selected based on three criteria. First, the typologies of the selected pottery fragments can still be identified according to their morphological features. Among the sampled pottery assemblage, six of which originally came from storing vessels (i.e., jars), eight sherds from cooking vessels (i.e., caldron, steamer, and tripod), and 27 from serving vessels (i.e., bowls). Secondly, we selected pottery sherd from different periods, namely from the Youziling, Qujialing, and Shijiahe Culture periods. The selection of samples retrieved from different occupation phases, including 10 potsherds from Phase I, 19 from Phase II, and 12 from Phase III (**Table 1**), allows an investigation of the potential chronological diet change at the Qujialing. Thirdly, pottery vessels from different contexts (e.g., from ash pits or stratigraphic layers, **Table 1**) were selected.

**Table 1:** Information of the studies pottery samples and the identified starch grains on the analyzed pottery sherds in the present study.

Lab No.	Simple. No	Simple Type	Date	job’s tear	millet	Unknowntuber	rice	acorn	Lotus	beans	Total
S1	2016HQQTN10W30④: S8	bowl	Youziling	0	8	4	0	0	0	0	12
S2	2016HQQTN10W30④: S9	bowl	Youziling	59	0	3	1	0	0	0	63
S3	2016HQQTN10W30④: S10	bowl	Youziling	0	38	23	0	0	1	0	62
S4	2016HQQTN10W30④: S11	bowl	Youziling	0	6	10	2	0	1	0	19
S5	2016HQQTN10W30④: S12	jar	Youziling	121	84	74	49	0	0	0	328
S6	2016HQQTN10W30④: S15	steamer	Youziling	35	5	12	10	0	8	0	70
S7	2016HQQTN10W30⑤: S18	bowl	Youziling	64	0	26	4	0	2	0	96
S8	2016HQQTN10W30⑤: S21	bowl	Youziling	13	0	1	0	0	3	0	17
S9	2016HQQTN11W30④: S26	tripod	Youziling	0	0	2	0	1	2	0	5
S10	2016HQQTN11W30④: S27	jar	Youziling	6	0	0	2	0	6	0	14
S11	2016HQQTN10W30②:S1	tripod	Qujialing	9	0	0	0	0	0	0	9
S12	2016HQQTN10W30②:S2	tripod	Qujialing	6	1	2	19	0	0	0	28
S13	2016HQQTN10W30②:S3	tripod	Qujialing	0	0	0	0	0	1	0	1
S14	2016HQQTN10W30③:S4	bowl	Qujialing	16	2	0	1	0	2	0	21
S15	2016HQQTN10W30③:S5	bowl	Qujialing	29	0	4	15	1	1	2	52
S16	2016HQQTN10W30③:S6	bowl	Qujialing	0	1	0	26	0	1	0	28
S17	2016HQQTN10W30③:S7	jar	Qujialing	8	0	2	0	0	0	0	10
S18	2017HQQTN49W03③:S32-34	caldron	Qujialing	0	12	1	0	0	0	0	13
S19	2016HQQTN13W30③:S40	bowl	Qujialing	21	10	11	8	0	1	0	51
S20	2016HQQTN13W30③:S42	jar	Qujialing	0	23	4	0	0	1	0	28
S21	2016HQQTN13W30③:S43	bowl	Qujialing	0	14	2	1	0	0	0	17
S22	2016HQQTN13W30③:S44	bowl	Qujialing	0	7	3	2	0	2	0	14
S23	2016HQQTN12W30④:S47	bowl	Qujialing	0	0	1	21	45	0	0	67
S24	2016HQQTN12W30④:S48	bowl	Qujialing	0	16	2	4	0	2	0	24
S25	2017HQQTN49W03③:S56	tripod	Qujialing	26	2	3	1	0	0	3	35
S26	2017HQQTN49W03③:S94	jar	Qujialing	1	0	0	0	0	4	0	5
S27	2016HQQTN88E17①:S102	caldron	Qujialing	19	0	1	25	0	3	2	50
S28	2016HQQ ash pit, 2⑤:S144	bowl	Qujialing	11	8	3	7	0	21	0	50
S29	2016HQQ ash pit, 2⑤:S155	bowl	Qujialing	0	1	0	3	0	7	0	11
S30	2016HQQTN25W41⑨:S62	bowl	Shijiahe	0	0	3	8	0	1	0	12
S31	2016HQQTN25W41⑨:S63	bowl	Shijiahe	0	4	0	0	0	0	0	4
S32	2016HQQTN25W41⑨:S64	bowl	Shijiahe	16	0	0	0	0	0	0	16
S33	2016HQQTN25W41⑨:S66	bowl	Shijiahe	0	5	0	2	0	0	0	7
S34	2016HQQTG8③:S74	bowl	Shijiahe	0	1	0	0	0	0	0	1
S35	2016HQQTG8③:S75	jar	Shijiahe	18	5	0	3	0	2	0	28
S36	2016HQQTG8③:S76	bowl	Shijiahe	0	0	0	0	0	0	0	0
S37	2016HQQTN25W41⑩:S90	bowl	Shijiahe	0	3	0	1	0	22	0	26
S38	2016HQQTN09W39H28:S126	bowl	Shijiahe	0	0	0	0	0	16	0	16
S39	2016HQQTN10W38③:S128	bowl	Shijiahe	83	25	20	7	0	21	1	157
S40	2016HQQTN09W39③:S130	bowl	Shijiahe	10	0	0	34	0	2	0	46
S41	2016HQQTN25W41⑨:S213	bowl	Shijiahe	6	0	0	0	0	1	1	8
			Total	577	281	217	256	47	134	9	1521
S42	control sample	soil from the site		0	0	0	0	0	0	0	
S43	control sample	water from the Lab		0	0	0	0	0	0	0	
S44	control sample	soil attached on pottery		0	0	0	0	0	0	0	

The chosen pottery sherds were sealed in separate Ziplock bags in the field laboratory and then transported to the Archaeobotany Laboratory at the University of Science and Technology of China (USTC) for further analysis. First of all, each of the pottery sherds was briefly rinsed with a wash bottle with ultra-purified water. Then, liquid samples from the internal surfaces of the pottery sherds were collected using an ultrasonic toothbrush. These liquid samples were gathered in test tubes for subsequent extraction. The liquid samples were treated with 10% HCl and 5% (NaPO_3_)_6_ to deflocculate clay minerals and other minor components, and then centrifuged in the presence of heavy liquid (CsCl with a density of 1.9 g/cm^3^) before being mounted on glass slides in a solution of 50% glycerine and 50% distilled water.

During the sampling process, extreme care was taken to avoid contamination at all stages, including the use of disposable powder-free gloves and disposable pipette suction heads. Additionally, we evaluated three control samples, including one soil sample taken from the archaeological site, one soil sample attached to the external surfaces of the pottery sherds, and the water used in the laboratory at USTC. The processes for processing the control samples were the same as the liquid samples taken from the internal surfaces of the pottery sherds.

The starch grains were examined with a Leica DM 4500P automated light microscope. Each slide was scanned in horizontal transects using a magnification of 400× under cross-polarized light. When starch grains were recognized by their extinction crosses, they were then further examined under the lens of 630×, with both brightfield and cross-polarized light. Two digital photographs (one under brightfield and one cross-polarized) were taken of each grain using a Spot Flex Mono 15.0 digital camera and Zeiss Axiovision software. The size of the starch grains was measured using AxioVision Rel. 4.7 software.

**Figure 4:**
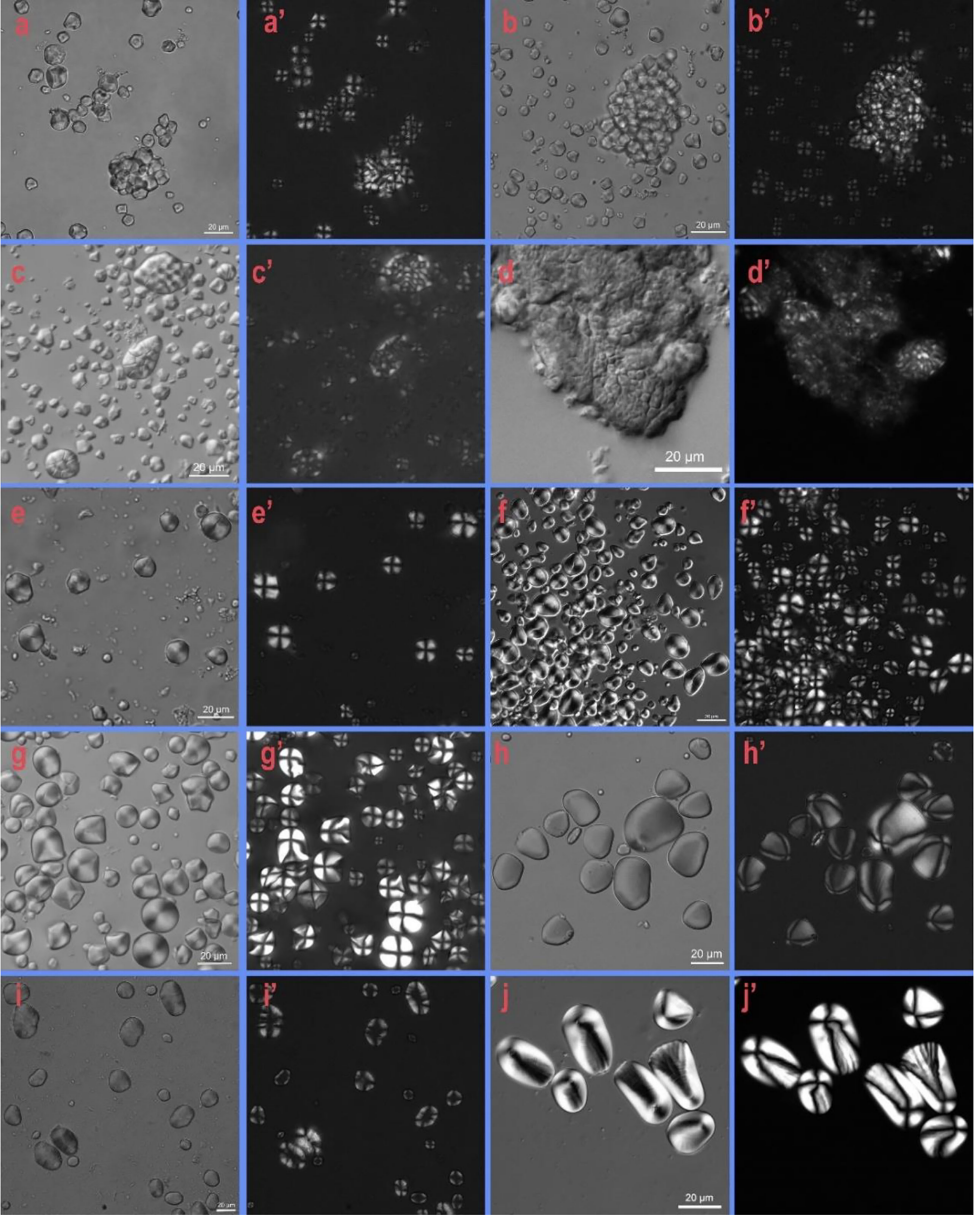
Examples of starch grain morphology from the modern reference (scale bar: 20 μm): (**AA’)** foxtail millet (*Setaria italica*); **(BB’)** broomcorn millet (*Panicum miliaceum*); **(C, D’)** rice (*Oryza sativa*); **(EE’)** job’s tears (*Coix lacryma-jobi*); **(FF’)** acorn **(***Quercus acutissima*); **(GG’)** snake gourd (*Trichosanthes kirilowii)*; **(HH’)** Chinese yam (*Dioscorea polystachya*); **(II’)** bean (*Vigna adenantha*); **(JJ’)** lotus root (*Nelumbo nucifera*).

The identifications of starch grains were based on modern starch references at the Archaeobotany Laboratory of USTC (e.g., **Figure 4**). We also referred to other published starch identification information, especially archaeological studies conducted in China, experimental work, and those focus on identifying modern starch samples (Henry et al., 2009; Wan et al., 2011; Wan et al., 2012; Liu et al., 2014; Yao et al., 2016).

## Results

A total of 1521 starch grains were extracted from 41 pottery samples from the Qujialing site (**Table 1**). No starch grains were detected in control samples taken from the laboratory, the site, and soil attached to the surfaces of the pottery sherds (**Table 1**), suggesting the most likely cause for entrapping the discovered starch grains was through intense or prolonged use of the pottery vessels as food-related implements in the past. Based on their sizes and morphologies, these starch grains were classified into seven distinct groups, including rice, millets (*Setaria italica* or/and *Panicum miliaceum*), job’s tears (*Coix lacryma-jobi*), acorns (*Quercus* sp.), beans (Fabaceae), lotus root (*Nelumbo nucifera*), and other types of underground storage organs (USO) possibly from Chinese yam (*Dioscorea panthainca*).

### Type I, rice

**Figure 5:**
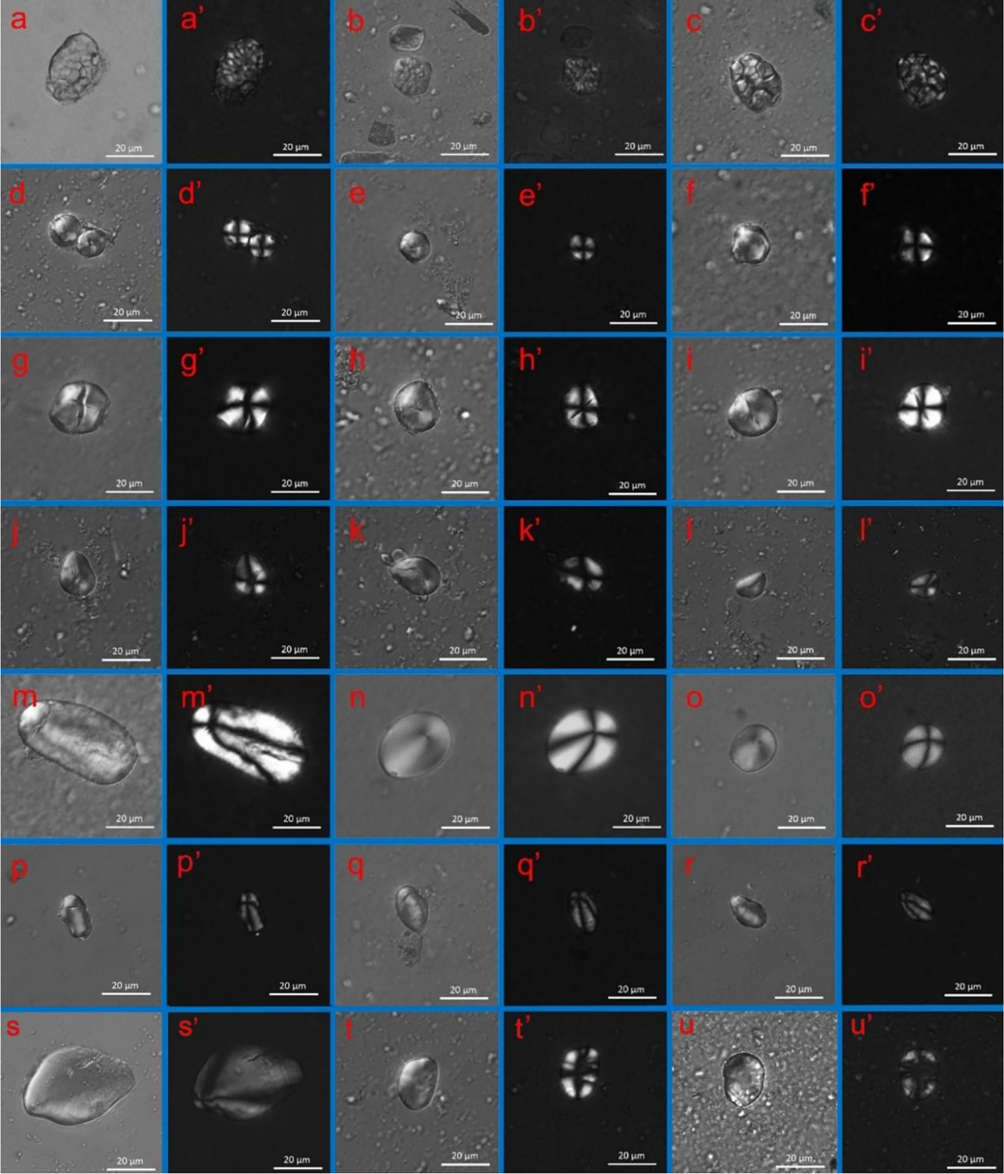
Starch grains retrieved from Qujialing Pottery samples under DIC and polarized filters (scale bar: 20 μm): **(A-C’)** rice (*Oryza sativa)* **(D-F’)** millet (*Setaria italica or/and Panicum miliaceum*)*;* **(G-I’)** job’s tears (*Coix lacryma-jobi*); **(J-L’)** acorn (*Quercus* sp.)*;* **(M-O’)** lotus root (*Nelumbo nucifera*); **(P-S’)** *starch grains from underground storage organs*; **(T-U’)** bean (*Vigna* sp. *or/and Vicia* sp.).

Starch grains from type I (n = 256) are polyhedral or round polyhedral in shape with the centric hilum closed, and they are common for the multigrain aggregation form (**Figures 5A-C’**). Each starch grain from Type I exhibited an extinction cross under polarized light, without visible fissures or lamellae. The extinction cross is shaped like an “X”, and the range of the individual particle sizes is 5.738–9.13 µm. Both the morphological characteristics, the particle size range, and the special compound starch grain structure coincide with starch grains from rice.

### Type II, millet

Starch grains of this type (n = 281) (**Figures 5D-F’** **)** are comparable with the subfamily *Panicoideae* in the Poaceae grass family. They have a polyhedral shape with angular or round edges, the hilum is centric, and fissures are fairly common, appearing as stars with a radiating. The extinction cross is generally “+” in shape, and very few of the crosses show a twisted pattern at the end of the arm. Lamellae are always invisible. The size range is 6.38–23.23 µm. They are similar in morphology to foxtail millet (11.21–16.75 μm) and broomcorn millet (5.92–13 µm) in our reference collection. Meanwhile, it has been noticed that starch grains from foxtail millet and broomcorn millet cannot be fully separated because of their similar morphological features (Yang et al., 2005; Yang et al., 2010; Liu et al., 2014). On the other hand, based on the morphological features of phytolith, it is possible to identify millet to a species level (Weisskopf and Lee, 2016). At the site of Qujialing, phytolith analysis has provided firm evidence that broomcorn millet and foxtail millet both existed (Yang et al, 2020), thus complementing our findings and interpretations.

### Type III, job’s tears

Starch grains of type III (n = 577) are polyhedral or oval-spherical in shape, exhibit Y-, V-, or linear-shaped fissures, centric or/and eccentric hilum, invisible lamellae, and have extinction crosses with straight or Zig-Zag arms **(** **Figures 5G-I’** **).** Starch grains from Type III are characterized by “Z” shaped arms on the extinction cross, a unique feature of job’s tears (Liu et al., 2014). The size range of type III grains is 6.87–26.780 µm, falling in the range of modern starch grains of job’s tears.

### Type IV, acorns

Starch grains of type VI (n = 47) are triangle ovate or water-drop shaped, with a centric hilum with an “×” shaped slightly bent extinction cross that appears under polarized light (**Figures 5J-L’** **)**. Fissures and lamellae are not visible. The size range is 9.74–24.93 µm. These starch grains resemble the starch grains from seeds of *Quercus* in the Fagaceae family, according to our previously documented work (**Figure 4**).

### Type V, lotus roots

Starch grains of type V (n = 134) are relatively large, showing subspherical or elongated oval shapes (**Figures 5M-O’**). The hilum is extremely eccentric and fissured with well-defined lamellae. The long axis length size range is 14.09–67.81 μm. There are wrinkles on the surface of the large grains, which is a particular characteristic of starch grains from lotus roots (**Figure 4**).

### Type VI, other USOs

starch grains of type III (n = 217) are round quadrilateral or elongated oval in shape, with extremely eccentric hila, visible lamellae in most cases, and extinction crosses with bent arms (**Figures 5P-S’**). The size range is 17.43-23.08 µm. Some of these particles show strong similarities in morphology and size with Chinese *yam* (*Dioscorea polystachya).* However, some starch grains from Type VI do not fully match the existing modern plant starch references, so we cannot exclude the possibility that they may come from other plant species.

### Type VII beans

Starch grains of type VII (n = 9) are elliptical or nearly kidney-shaped starch grains that have radiating fissures with clear-cut lamellae (**Figures 5T-U’**). Under polarized light, the extinction cross of starch grains from type VII resembles two tangent curves. The size range is 15.84–45.23 µm. According to our modern starch grain database (e.g., **Figure 4**), it is very difficult to separate starches from different beans according to their morphological features. Although it has been proved that the compositional and physiochemical properties of starches from different beans vary (Zhang et al., 2018), the results can hardly be applied to archaeological research because the identifications of archaeological starch grains are largely based on their morphological features under light microscopy. Thus, we avoid over-interpret type VII starches to a precise taxonomy level, as they may include *Vigna* sp. or/and *Vicia* sp.

## Discussion

### New insights into the edible plant foods at Qujialing

**Figure 6:**
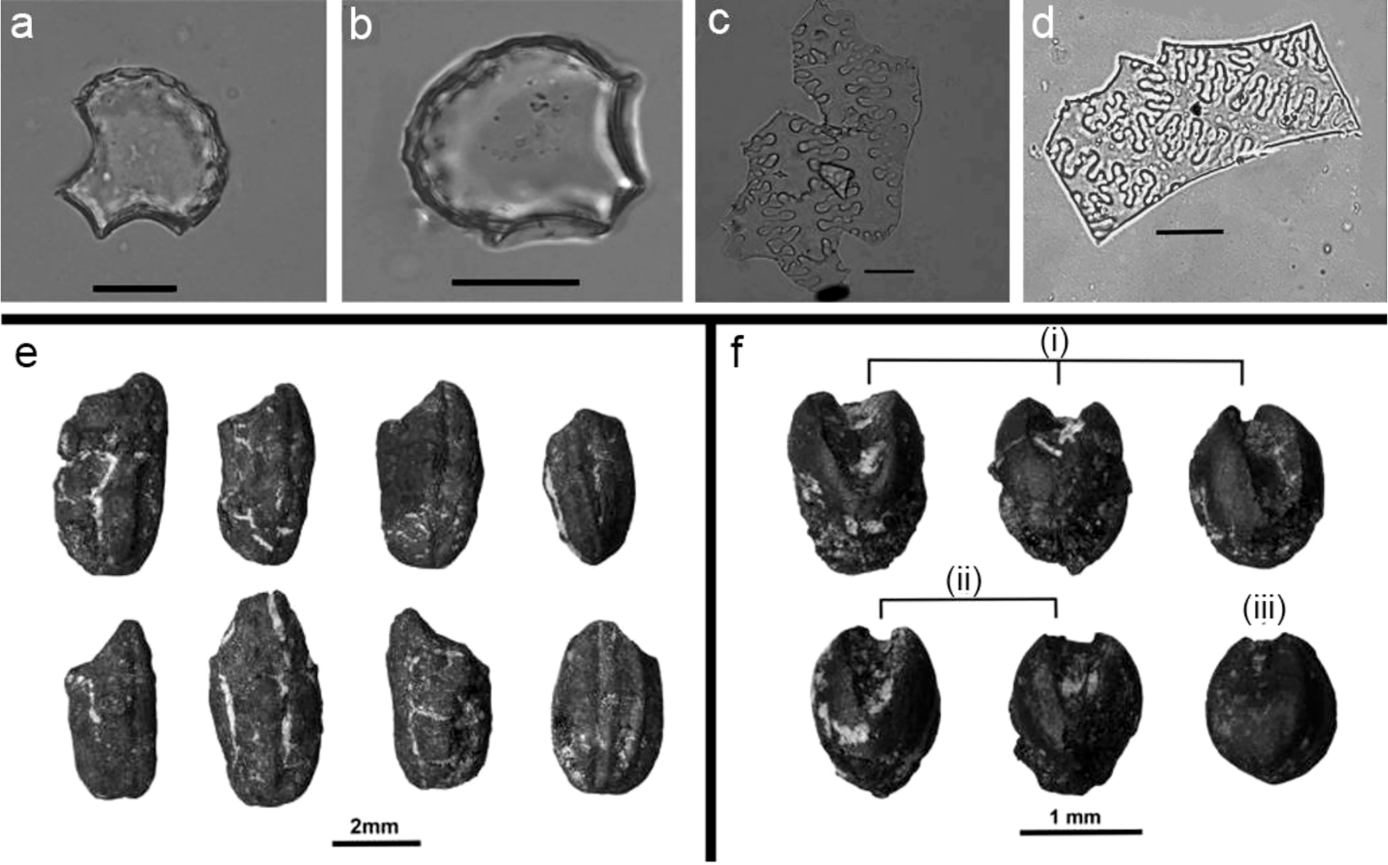
Phytoliths and macrofossil remains from rice and millet at the site of Qujialing (After Yao et al., 2019; Yang et al., 2020). **A, B**: cuneiform bulliform phytoliths from rice; **C**: Ω-undulated type, endings structures of epidermal long cell from foxtail millet; **D**: η-undulated type, endings structures of epidermal long cell from broomcorn millet (scale bar for figure 6a-d: 20 mm); **E**: macrofossil remains from rice during the Youziling Culture period; **F** (i) macrofossil remains from foxtail millet during the Youziling Culture period; **F** (ii) macrofossil remains from foxtail millet during the Qujialing Culture period; **F** (iii) macrofossil remains from foxtail millet during the Shijiahe Culture period.

Based on our previous floatation work at Qujialing, macrofossil remains from rice (**Figure 6E**), foxtail millet (**Figure 6F**), soybean (*Glycine max*), other types of beans (*Leguminosae*), acorns (*Quercus* sp.), jujube (*Ziziphus* sp.), persimmon (*Diospyros* sp.), plum (*Armeniaca mume* Sieb.), water chestnut (*Trapa* sp.), and Gorgon fruit (*Euryale ferox* Salisb.) were found (Yao et al., 2019). In addition, phytoliths from rice (**Figure 6A and 4B**), foxtail millet (**Figure 6C**) and broomcorn millet (**Figure 6D**) were identified in the soil samples taken at Qujialing (Yang et al., 2020). These findings reveal great available plant food resources at Qujialing, including not only crops such as rice and millet, but also beans, nuts, and various fruits.

**Figure 7:**
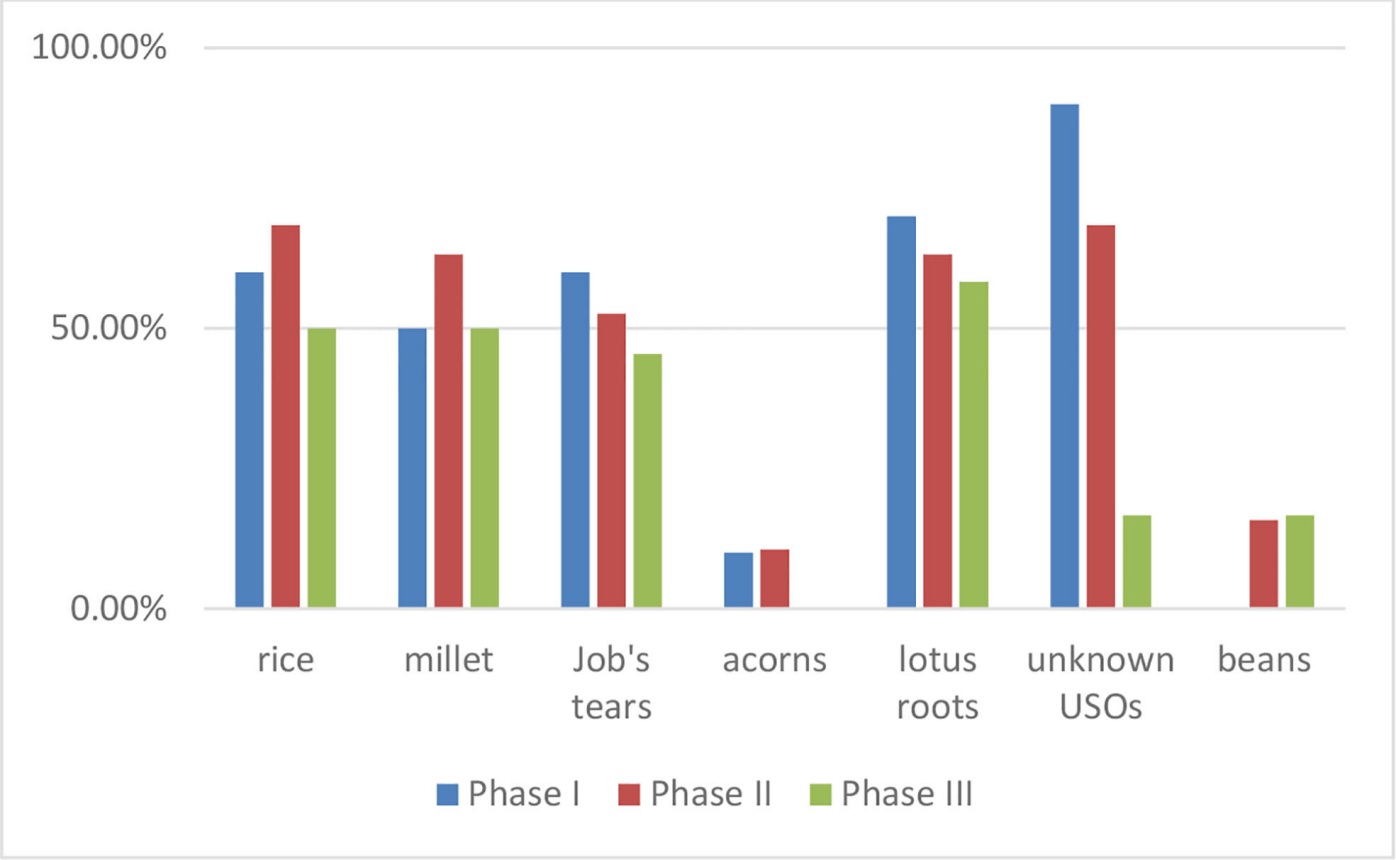
Ubiquities of different types of starch grains on Qujialing pottery vessels attributing to the different occupation phases.

Apart from fruits, the majority of the above-mentioned starchy foods have been discovered on the pottery vessels from Qujialing in this study, suggesting the ancient Qujialing people took advantage of their local plant recourses. Notably, job’s tears and lotus roots were not discovered in the previous archaeobotanical work at Qujialing. These new findings in the current study thus complement the previous archaeobotanical work, providing direct evidence that job’s tears and USOs were among the plant foods that were consumed by the ancient Qujialing people (**Figure 7**).

Following the criteria that have been established for the identification job’s tears and other related plant species (Liu et al., 2014), the study presents here successfully identified the starch grain from job’s tears. Job’s tears have a wide geographic distribution in China and are generally used for foods and traditional Chinese medicine. On the Qujialing pottery vessels, the ubiquity of job’s tears appeared more frequently than in rice and millet (**Figure 7**). Nevertheless, neither of the macrofossil or phytolith from job’s tears have been discovered in our previous studies at the site of Qujialing. In a previous study, a discrepancy between the findings regarding macrofossil and microfossil of job’s tears at Chinese prehistoric sites has been noticed (Liu et al., 2019). So far, macrofossil remains from job’s tears have only been reported at three Neolithic sites along the Yangtze River, namely the sites of Baodun (Guedes et al., 2013), Chengtoushang (Liu, 2007), and Hemudu (Zhejiang Institute of Archaeology, 2003) in the upper, middle, and lower catchment of Yangtze River respectively. In contrast, starch grains from job’s tears have been discovered on pottery vessels or grinding tools unearthed from over 30 sites across China (see the summary by Liu et al, 2019). Thus, Liu and colleagues (2019) have investigated this issue with a multidisciplinary approach and described the possible pathways of job’s tears, from its cultivation, and processing, to the later stages of post-depositional processes. Because the edible part of job’s tears (i.e., caryopsis) is covered with thick utricles, so the caryopsis very likely ended up in small pieces after hulling. Thus, it has been proposed the fragments of caryopsis and utricles from the processed job’s tears were more difficulted to be discovered and identified. In terms of phytoliths from job’s tears, which show a great variety of types, were difficult to be identified, until the recently published methodology (Duncan et al., 2019).

In addition, starch grains from lotus roots have been identified on the pottery vessels, which had not been discovered at Qujialing according to evidence from phytolith or macrofossil remains (Yao et al., 2019; Yang et al., 2020). Lotus root is rich in starch, protein and other nutrients. Since the early Neolithic period, lotus roots were utilized by the Chinese ancestors, especially at the sites that were located near perennial water sources (Zhao and Zhang, 2009; Yao et al., 2016). Previous archaeological exploration at Qujialing revealed that two rivers, namely the Qingmudang River and the QingPu River, run alongside the site (Tao et al., 2019). Such an environment was ideal for lotus roots to grow, providing an important supplementary food source for the ancient Qujialing people. In the research region today, lotus roots are always on the menu at the local restaurants. The old saying “no soup, no banquet” in the Hubei Province describes how much Hubei natives love their soup, a special dish cooked with pork rib and lotus root. This specific dietary habit of the local people could have been inherited from their ancestors, considering the prolonged exploitation and consumption of lotus root at Qujialing and other sites along the catchment of the Yangtze River (Yao et al., 2016).

It also worth noting that charred remains from soybeans (*Glycine max*) dated to the Eastern Zhou Dynasty (770-256BC) were identified at Qujialing (Yao et al, 2019), while starch grains from beans were discovered on pottery vessels attributed to the Phase II and Phase III at the site. The starch data thus extends the record of consumption of beans at the site of Qujialing by around 2000 years. Although legumes play an important role in Chinese food and agriculture nowadays, macrobotanical remains from legumes have not been reported in the previous floatation work conducted at the Neolithic sites in the Hubei Province (e.g., Deng et al, 2013; Tang et al, 2014; Tang et al, 2017). The quantity and ubiquity value of legumes are both low at the site of Qujialing (**Figure 7**). These results imply that legumes probably were not regularly used in the Neolithic Hubei Province.

### Neolithic dietary choices towards rice and millet

The results from starch grain analysis on pottery vessels suggest that both rice and millet were consumed at the site of Qujialing, consolidating our previous work based on macrofossil remains and phytoliths. Based on the data from the floatation work, the total identified number of macrofossil rice remains from the prehistoric period at Qujialing are highest (n=528) among other types of cereals (Yao et al., 2019). During the first two occupation phases at Qujialing, the identified rice macrofossil remains account for the largest percentage among other species (82.94% and 82.26% accordingly), then dropped to 45.10% during Phase III. The ubiquity of rice macrofossil remains also went down from 82.61% in the Youziling Culture period, to 69.40% and 63.63% in the latter two occupation stages. Furthermore, the results from phytolith analysis echo the findings based on macrofossil remains, revealing that the number of phytoliths from rice decreased slightly from 11.9% in Phase I to 10.57% in Phase II, and then to 7.58% in the last occupation phase at Qujialing (Yang et al., 2020). The starch data also reveals the ubiquity of starch grains from rice was also the lowest in the Shijiahe Culture period (**Figure 7**), which is consistent with the findings based on macrofossil remains and phytoliths.

In terms of millet, the earliest remains of this plant have been recovered on pottery sherds at grinding tools at the sites of Nanzhuangtou (ca. 9.500-9.000 BC) and Donghulin (ca. 9000-7500 BC) in the upper Yellow River valley, as attested by starch grain analysis (Liu et al., 2010b; Yang et al., 2012). Then, millet farming had spread southwards and reached the catchment of Han River and Liyang Plain by 6000 years ago (Nasu et al., 2007; Fu et al., 2010; Deng et al., 2015; Weisskopf et al., 2015), corresponding to the Phase I at Qujialing.

The yielded starch data shows that millet had been consumed since the Youziling Culture period and its ubiquity was even close to rice starches on the pottery vessels, although never exceeded (**Figure 7**). Differently, according to the previous archaeobotanical work at Qujialing, millet less likely surpassed rice and became the predominate type of crops at Qujialing (Yao et al., 2019). The ubiquity of macrofossil remains from foxtail millet increased from 6.52% in the Youziling Culture period to 27.80% and 18.18% in the latter two occupation phases at Qujialing. Moreover, much fewer macrofossil remains from foxtail millet (n=27) and broomcorn millet (n=0) were discovered during our previous floatation, in contrast to a large amount of macrofossil remains from rice (n=528). Similarly, only a few (n ≤ 6) phytoliths from foxtail millet and broomcorn millet were discovered in soil samples taken from the site of Qujialing, while hundreds of phytoliths from rice were identified (Yang et al., 2020). The results yielded from macrofossil remains and phytoliths thus seem to contradict the result from starch grain analysis. Nevertheless, it should be noted that starch grains from rice are small (normally less than 10 µm) and difficult to be discovered and identified in the archaeological samples (Liu et al., 2010c; Yang et al., 2015; Li et al., 2020a), which could have led to a biased interpretation of starch data.

Bearing this factor in mind and taking into account the previous archaeobotanical data, we suggest that the ancient Qujialing people possibly consumed more rice than millet. Later, even when wheat (*Triticum aestivum*) had also been introduced into this region since at least the Warring States Period (Yao et al., 2019), rice remains the main type of staple food in the Jianghan Plain today. The dietary tendency to rice could be related to the local dietary habits, considering the middle catchment of Yangtze River had a long history of rice cultivation. In the middle catchment of Yangtze River, previous phytolith studies at the palaeolithic site of Diaotonghuan indicated that wild rice grew there and was exploited by local people after 12, 000 BP (Zhao, 1998). In the later Pengtoushan Culture (7500-6100 BC), thousands of macrofossils from rice were found. The size of the Pengtoushan rice was larger than the local distributed wild rice (Crawford and Shen, 1998; Wang et al., 2010), providing more solid evidence for rice domestication in this area. Afterwards, although millet appeared at Qujialing and other nearby contemporary sites, rice remains were always more common (Luo et al., 2019a).

### Dietary trends at Qujialing

According to the further analysis of the yielded starch data (**Figure 7**), the ubiquity of starch grains from agricultural products (i.e., rice and millet), job’s tears, and lotus root are high (over 50%) and relatively the same in different occupation phases at Qujialing. Differently, starch grains from the unknown USOs and acorns changed more dramatically from Phase I to Phase III. In Phase I and Phase II, the ubiquity of the unknown type of USOs were above 50% but decreased to around 16% in Phase III (**Figure 7**). Similarly, in Phase I and Phase II, the ubiquities of acorns are 10% and 10.53% respectively. In Phase III, starch grains from acorns are no longer present on the pottery vessels. The macrobotanical remains from acorns also account for a small percentage, with low total quantity (n=11) and ubiquity (less than 10% in each phase) (Yao et al., 2019).

Palynological analysis of both sedimentary profile and cultural layers at the site of Qujialing indicates that acorns (*Quercus Deciduous* and *Quercus Evergreen*) distributed in the area 5400-4200BP (Li et al., 2009), and the earliest exploitation of acorns dates back to the upper Palaeolithic period in China (Yang et al., 2009; Liu et al., 2011). Afterwards, macro- and micro-remains from acorns were continuously found at numerous early and middle Neolithic sites in southern and northern China (Liu et al., 2010a; Liu et al., 2015; Liu, 2015; Yao et al., 2016), indicating acorns were important sources of plant foods during that time. Because acorns were more likely procured through gathering rather than cultivation, archaeological findings of extensive acorn leftovers on grinding tools and pottery vessels were only used as indicators for a broad-spectrum subsistence economy (Liu et al., 2010a). With the overview of the previous archaeobotanical work in China, it has been proposed that agriculture has already become the main subsistence strategy in the middle catchment of Yangtze River since the period from 6300 to 5300 BP (Zhao, 2009), corresponding to the research period in the current study. Therefore, the more developed agriculture at Qujialing, especially in Phase III, could have contributed to the gradual replacement of acorns with other agricultural products (i.e., rice and millet) and let acorns become less important in Qujialing people’s diet. This proposition could also explain the decrease in the ubiquities of the unknown type of USOs found in this study.

Previous archaeological studies focus on dietary trends and choices often illuminate ancient interactions between society and nature and reflect diverse cultural traditions in different communities (Dong et al., 2021). The dietary trends at Qujialing, characterized by less consumption of non-agricultural crops (i.e., acorns), were very likely resulted from the development of agriculture in the research region.

## Conclusion

The identifications of starch grains discovered on potsherds attributing to different occupation phases at Qujialing, for the first time, provide valuable information regarding prehistoric consumed plant foods in the Jianghan Plain in the middle catchment of Yangtze River. Apart from rice and millet that were previously identified based on macrofossil and phytolith analysis, starch grains from other species including job’s tears, beans, acorns, lotus roots, and other tubers (perhaps Chinese yams) were detected on the Qujialing pottery vessels. The starch data thus consolidates the previous archaeobotanical work based on macrofossil and phytolith analysis at Qujialing, and put more new insights into the plant foods that had been consumed by the local people during the Neolithic period. Further quantitative analysis of the yielded data from different analytic methods, namely starch grain, phytolith, and macrofossil analysis, indicate that although millet had spread into the Jianghan plain since the earliest occupation period at Qujialing, rice had always been the predominate cultivated crop and probably more frequently consumed. Specific dietary choices and trends were also detected at Qujialing, including persistent exploitation of lotus roots throughout the site occupation and the abandonment of acorns in Phase III, which may be related to the local environment that was surrounded by water and developed agriculture accordingly. Yet, apart from the present research, starch grain analysis has rarely been applied to investigate the uses of Neolithic pottery vessels in the Jianghan Plain, more future work thus is still needed to deliver a more comprehensive understanding of the past human diet in the whole research region.

## Data availability statement

The original contributions presented in the study are included in the article/supplementary material. Further inquiries can be directed to the corresponding authors.

## Author contributions

YY, WL and JZ conceived and designed the study. YT, DZ, and YL provided the archaeological samples. MK and LY collected the study samples and analyzed the data. WL, YY, and MK wrote the manuscript. All authors contributed to the article and approved the submitted version.

## Acknowledgments

This work was supported by the International Partnership Program of the Chinese Academy of Sciences(132311KYSB20190008), the National Natural Science Foundation of China (41772172), the Philosophy and Social Science Planning Project of Anhui Province (Grant No. AHSKQ2021D52), the USTC Funding for Featured Liberal Arts (YD2110002016), and the USTC Youth Innovation Fund (WK2110000020). We would like to thank three reviewers for their insightful comments.

## Conflict of interest

The authors declare that the research was conducted in the absence of any commercial or financial relationships that could be construed as a potential conflict of interest.

## Publisher’s note

All claims expressed in this article are solely those of the authors and do not necessarily represent those of their affiliated organizations, or those of the publisher, the editors and the reviewers. Any product that may be evaluated in this article, or claim that may be made by its manufacturer, is not guaranteed or endorsed by the publisher.
